# Experiences of living with type 2 diabetes in Pakistan: the role of culture and family in physical activity

**DOI:** 10.1186/s12939-022-01706-4

**Published:** 2022-07-29

**Authors:** Omama Tariq, Claire Rosten, Jörg Huber

**Affiliations:** 1 Institute of Applied Psychology, University of the Punjab, Lahore, Pakistan; 2 School of Sport and Health Sciences, University of Brighton, Brighton, UK

**Keywords:** Type 2 diabetes, Physical activity, Exercise, Lifestyle, Family, South Asian culture

## Abstract

**Background:**

Diabetes-related guidelines recommend lifestyle changes for people living with type 2 diabetes (PLwD). In South Asian (SA) families, cultural and contextual expectations often influence people’s decisions. However, broad explanations provided in the existing literature and theories concerning family involvement can increase the chance of health professionals overlooking the complexities of family roles within SA communities. Previous literature has identified the need to examine the perspectives of PLwD and their family members in Pakistan to shed light on factors perceived to support and hinder recommended physical activity (PA) to manage type 2 diabetes. This study explored (1) the enablers of and barriers to PA in the context of PLwD in Pakistan and (2) family involvement regarding PLwD’s engagement with PA.

**Methods:**

Semi-structured interviews were conducted with 30 PLwD and 17 family members of PLwD who were recruited in metropolitan Lahore (Pakistan) and primarily used state health services available to relatively disadvantaged populations. Interviews were transcribed and analysed using thematic analysis.

**Results:**

Three themes were identified: (1) Going for a walk as a feasible PA; (2) the role of family members in influencing PA; and (3) gender differences and cultural acceptability of an activity. PA enablers for PLwD consist of gender-specific opportunities for activity facilitated by peers and family members. Culturally acceptable opportunities for PA in Pakistan for specific genders and age groups within the socio-cultural context constituted an essential factor. In this study, all women with diabetes described walking as the only acceptable form of PA, whereas some men mentioned other activities such as running, playing cricket, and cycling.

**Conclusions:**

Medical guidelines must consider patients’ daily routines, account for cultural and familial expectations of different genders and age groups, and address social and physical barriers encountered by these different groups to encourage PA among PLwD in SA cultures.

## Background

*Type 2 diabetes mellitus* (T2DM) has become a leading cause of disability worldwide [1]. In recent years, 41 million deaths occurred annually due to non-communicable diseases (NCDs) in low- or middle-income countries (LMIC) [2]. As in the case of other LMICs, Pakistan faces the burden of an increase in T2DM. The International Diabetes Federation [3] reported the number of people living with type 2 diabetes (PLwD) (20–79 age range) in South Asian (SA) countries as follows: Pakistan, over 7 million; India, 69.1 million; and Bangladesh, 7.1 million. However, the statistics reported do not separate the cases of type 1 and type 2 diabetes, making it challenging to identify the exact number of T2DM cases [4]. Aamir et al. [5] reported that the prevalence of T2DM across Pakistan was 17%. The number of cases in Pakistan is expected to rise to 13.8 million in 2030, raising concerns and suggesting a need to explore diabetes management and control [6]. One reason for the increased prevalence of T2DM in SA countries, such as Pakistan, is a carbohydrate-rich diet and physical inactivity [7].

Like many other countries, Pakistan has an NCD unit within the Ministry of Health. However, it does not have a strategy to reduce physical inactivity [8]. Moreover, in Pakistan’s public sector, there is a three-tiered healthcare delivery system: primary care facilities for outpatients (basic health units and dispensaries), secondary district hospitals that provide basic care for PLwD and outpatient care, and tertiary care hospitals in urban areas [9, 10]. There is no specific care pathway prescribing diagnostic and therapeutic processes for PLwD in Pakistan. For access to doctors and medical treatment, PLwD can visit private clinics or government-based outpatient clinics [11]. In the private sector, clinics, hospitals, and NGOs provide services to PLwD [8, 12]. Some organisations focus on educating people about diabetes [8, 13]. It is unclear what impact these educational efforts have due to a dearth of literature assessing their effectiveness.

The World Health Organization’s “Global Action Plan for the Prevention and Control of NCDs 2013–2020” aimed to motivate people to work towards an active lifestyle not only to reduce risk factors for diabetes but also to control the disease [14]. Limited financial resources make the investigation of potentially more cost-effective factors, such as enabling physical activity (PA) in PLwD, necessary.

The United Kingdom’s Department of Health and Social Care, through its Chief Medical Officer, recommends 30 minutes of exercise at least five times a week for PLwD, including brisk walking, exercising in a gym, or participating in sports [15]. Similarly, in Pakistan, the National Association of Diabetes Educators of Pakistan recommends walking for 30 minutes 5 days a week, along with a range of other exercises [16]. Physicians in Pakistan recommend walking to most PLwD because it is considered safe to engage in moderate-intensity walking [16].

Some of these guidelines may not be helpful in SA communities. There are multiple accounts in the literature of cultural disparities in SA communities regarding the choice of PA between men and women. A systematic review highlighted that women are less active than men [17]. A study in Pakistan reported walking as a common activity for both genders, but men were more likely to cycle than women, and walking was more culturally acceptable for women [7]. This disparity in choices in PA is unacknowledged in general guidelines provided by the National Association of Diabetes Educators of Pakistan [16]. The guidelines make similar general recommendations for men and women with diabetes (*a one-size-fits-all approach*).

Furthermore, few studies exist that explore factors influencing PA in Pakistan compared with studies conducted on Pakistanis living abroad [7, 18]. In one study [7], PLwD preferred walking a distance less than one mile. A gender difference was also seen; men under the age of 40 preferred to cycle (those over the age of 40 preferred to walk a distance of one to five miles), whereas women were reportedly more involved in household activities. Moreover, a study of Pakistani immigrants living in the United Kingdom showed that exercise is seldom regarded as a priority. Women especially reported inactivity. One reason was the unavailability of same-gender activities, as multi-gender activities conflicted with their religious beliefs [18]. This indicates that culture and religious beliefs can be barriers to engaging in PA. Imran et al. [19] describe these social pressures as cultural influences whereby social norms inform the acceptance of gender-specific behaviour for PLwD.

Many factors specific to Pakistani culture may influence PA. In Pakistan, a family is a close unit, more collectivistic [20, 21] than individualistic [22]. The family system can be nuclear or joint. The nuclear family consists of two generations (parents and children), whereas in a joint family, three or more generations live together, including grandparents, brothers and their wives, sisters and their husbands, uncles, aunts, and nephews/nieces [23]. Generally, the male members make decisions in household affairs or daily matters [24]. Certain behaviours are expected of each family member, and these vary across age and gender. Parents’ and children’s roles in Pakistani society vary. The father is seen as an authority figure, responsible for making decisions regarding the family and looking after family members [24]. Cultural family roles in Pakistan, as in other SA countries or collectivistic societies, are heavily influenced by tradition [23, 25]. Maintaining identity within these roles is important and may influence the amount of support provided, and control exerted, in the family towards different family members [23–26].

Families within a particular culture act as enablers regarding PA. For example, Samir, Mahmud, and Khuwaja [27] collected information regarding reasons for physical inactivity from obese family members accompanying patients at a hospital in Pakistan. They concluded that a lack of family support can be a barrier to engaging in PA. More than 50% of respondents reported that the absence of a family member accompanying them for PA, particularly a spouse or child, decreased their desire to exercise. They also showed that people living in a joint family system were more likely to be inactive compared to those living in a nuclear family system. Although the study does not provide reasons for this inactivity observed in joint family structures, it can be assumed that the presence of more people in a household may lead to fewer chores and therefore less PA for individual members. In addition, environmental barriers were also reported: an unsafe neighbourhood, lack of places to go, and lack of time.

Lawton et al. [28] explored experiences and perceptions of Pakistani and Indian people with T2DM in Edinburgh, United Kingdom, in relation to PA. They found that the choice by participants to perform PA was dependent on factors such as fulfilling family obligations, limiting the exposure of women in mixed gatherings, and a lack of sports activities. Similarly, family influences related to health aspects were seen in both SA people living abroad [29] and those living in their home countries; however, their influence, along with the cultural setting, was not considered a central management point. A study in Pakistan [30] showed that doctors regarded family members as important in managing the treatment of the patient. As the authors noted, *“There is no difference between the patient as an individual and his family. Both are one and the same*” (p. 94).

The broader societal and cultural context in which PLwD live is an under-explored area. Many studies on PLwD have focused on individualised education patterns [31] and self-regulatory practice for lifestyle changes [30, 32]. Taking a PLwD-centred and individualised Western approach may not be successful in a collectivistic culture [30]. A culturally appropriate programme of lifestyle modification requires sensitivity to people living in collectivistic societies. As Greenhalgh [33] report, a health programme that only targets meeting educational goals will not succeed as behaviours are also influenced by traditional and cultural beliefs.

People living in close relationships and in communities influence each other’s behaviour at a social level. This acts as an enabler if it facilitates lifestyle adjustments and a barrier if it does not. Therefore, different theoretical perspectives at various levels (from the individual to the societal) shed light on this study’s research questions by considering how personal beliefs, family involvement, and social contexts affect lifestyle adjustments: the common-sense model of illness [34] for individual-level health cognition, health-related social control [35] for that at the interpersonal level, and the Society-Community-Family model [19] for that at the social and cultural level.

This study aimed to understand the experiences of PLwD and their family members in Pakistan regarding PLwD’s PA engagement for managing type 2 diabetes. To this end, the following research questions were posed:

- How do PLwD incorporate doctors’ recommendations for PA to manage diabetes while living in Pakistan?
- What do PLwD and their family members perceive as enablers and barriers to PA engagement for managing type 2 diabetes while living in Pakistan?
- What strategies do PLwD or their family members employ to help PLwD engage in PA?

## Methods

### Study design

This qualitative study employed a single, semi-structured interview of each participant. Two separate interview guides were created, one for PLwD and the other for family members of PLwD. Semi-structured interviews were the data-collection method of choice due to their flexibility in allowing issues to be explored in detail [36–38]. This method adds depth to the understanding of issues pertaining to engaging in PA for PLwD in Pakistan.

The two interview guides for PLwD and their family members were developed based on Keogh et al. [39], Sullivan-Bolyai et al. [40], and the researchers’ reading of the relevant literature, informing the design of the interview guide exploring the experiences of PLwD. These guides were developed after reviewing the literature that provided the background to the conceptual framework of this study [39, 40]. The initial interview guide was pilot-tested and modified as needed (e.g. questions needing elaboration or use of different words). The interview guide for PLwD focused on their understanding of their condition followed by details of the PA they performed and how it influenced the management of their diabetes. Regarding PA, they were asked about what does and does not work for them and about their views on their family’s involvement with their PA. Similarly, the interview guides for family members of PLwD focused on their understanding of the diabetic family member’s condition. Regarding PA, they were asked about what had and had not worked for their family member with diabetes as well as their views on their family’s involvement with the PA of the PLwD.

#### Data collection: settings and participants

Participant recruitment started with purposive sampling and gradually progressed to snowball sampling. The sampling strategy was changed to increase the possibility of recruiting willing participants living within the same metropolitan region (Lahore). Recruitment sites were selected based on similarities in the characteristics of PLwD as provided by hospitals in Lahore. Moreover, the diabetes centres covered PLwD from a wider area than done by general practitioner clinics, which would limit the research not only to a single doctor but also to PLwD living in that locality. The sites are not named to maintain the confidentiality of the places where data were generated.

#### Sampling, recruitment, and consent

The participant inclusion criteria were as follows:

##### For PLwD

- Individuals with T2DM for at least 1 year’s duration, as they would be familiar with their condition; they would also be able to speak knowledgeably regarding their experiences living with diabetes (LwD) and regarding enablers of and barriers to the recommended PA.
- Individuals living with at least one adult family member; those living on their own would have different environmental factors influencing them than someone living with family members.
- Individuals aged 30 years and above, as the prevalence of T2DM in SA communities starts increasing at age 25.

##### For family members

- Individuals 18 years or older.
- Individuals living in the same household as the person LwD; it did not matter if the family member had a medical condition as long as it did not hinder their ability to support PLwD PA, and the individual had to be involved in the care of the person LwD.

### Ethics statement

This study was approved by the Ethics Committee of the University of Brighton. The recruitment sites granted permission. Participants provided informed consent before data collection.

### Sample description

Thirty PLwD (13 men, 17 women; 30–70 years) and 17 family members of PLwD (4 men, 13 women; 22–61 years) were recruited.

#### PLwD

The sample comprised 17 women (30–54 age group *n* = 10; 55+ age group *n* = 7) and 13 men (30–54 age group *n* = 6; 55+ age group *n* = 7). The majority were married (*n* = 26), a small number were widowed (*n* = 4), and around one-third had no formal education (*n* = 11). Most PLwD used medications (*n* = 21). People with diabetes were more likely to come from a joint family system (*n* = 18) in comparison to those coming from a nuclear family (*n* = 12). All respondents lived in an urban setting, and almost half were employed (*n* = 14).

#### Family members

Most of the 17 family members recruited were married (*n* = 11; single *n* = 6). The majority had some form of education (5th grade *n* = 2; 8th grade *n* = 2; 10th grade *n* = 1; 12th grade *n* = 4; 14th grade *n* = 3; master’s and above *n* = 3). There was an almost equal representation of participants from both types of family systems (nuclear *n* = 9; joint *n* = 8). All respondents resided in an urban setting, and most were unemployed (*n* = 11).

### Data analysis

Interviews were transcribed in Urdu, translated into English, and then analysed using thematic analysis [41]. The transcribed interviews were re-read to gain a better sense of the data (familiarisation). Initial thematic categories were drawn from various theoretical backgrounds and the literature. After the first 16 transcripts had been reviewed, the authors re-evaluated the codes and moved towards a data-driven approach that was more informative regarding the participants’ experiences and that was better able to answer the research questions. Similar codes were grouped to determine how they aligned in terms of the subthemes. The questions addressed were as follows:

- What happened, where did it happen, when did it happen, and why did it happen? The most important question was why, as it helped reveal patterns within the data.
- What made this different? Why were some changes easy for some PLwD but not for others?

The themes and subthemes did not remain fixed; they were refined while writing the results section. The subthemes needed further refinement to better understand what participants said in the interviews and answer the research questions. The results section thus went through multiple iterations. The material background helped clarify patterns and highlighted that a particular situation that was an enabler for some was a barrier for others based on their role within the culture.

## Results

Three themes were identified: (1) Going for a walk as a feasible PA, (2) the role of family members in influencing PA, and (3) gender differences and cultural acceptance of an activity. Quotes exemplifying the subthemes are presented in each subtheme’s description.

### Theme 1: going for a walk as a feasible PA

The findings of this study revealed that walking was a strong subtheme among PLwD and an important type of PA recommended to them. The first theme captured available options for activities acting as enablers and highlighted that walking was the preferred form of PA for PLwD.

#### Subtheme 1: walking as a common form of PA that is adjustable to people’s needs

All the PLwD and their family members mentioned walking as a form of PA for PLwD, and it was the dominant activity noted in the interviews.

*I do not do any exercise; I only walk as an activity.* (25, man LwD, age 55)

Our study found that PLwD and their family members perceived walking as the preferred PA, as it can be adjusted according to the situation, the health, and, for a few, the age of the PLwD.

*If someone accompanies me for morning prayers [to the mosque], I walk at his [peer’s] speed. If I go alone, I walk fast. When I walk up the street, on my way back, my speed gets slower.* (16, man LwD, age 46)

Walking was preferred because it is feasible for a variety of reasons. One such reason is that PLwD can easily adjust to and sustain this type of PA. Going for a walk could easily be adjusted in terms of pace according to the person’s routine, bodily ability, and age. For PLwD and their family members, walking was also perceived as safer and less intense than other types of PA.

*He is perfectly fine. He can walk for a longer duration. If you suggest that he starts running, he will have knee pains. He opts for activities that fit better with his age and ability.* (10, wife of man LwD)

Similarly, a small number of men LwD described their preference for walking over going to a gym:

*Look, walking is our exercise. If I go for one hour of walking, I treat the walk as exercise. I do take normal walks. I don’t get a lot of time. And it is pointless, what do you call those places of working out … ?*

*Interviewer: The gym?*

*Gym, I don’t like to go there.*

*Interviewer: Why?*

*I do not want to be a bodybuilder. I do not want to lose weight. My weight is normal. My weight is around 70 [kg]. I think it’s a good range. Why would I need to increase the weight to 80/90 [kg] or reduce it to 60 [kg]? I do not need it*. (13, man LwD, age 68)

This respondent described how he perceived that the reason a person would go to a gym would be to build muscle, gain strength, or lose weight. Although PLwD should aim to lose weight, the respondent had an average body weight. This illustrates that perceived use of a PA influences engagement with that activity.

Women were found to be even less likely to go to a gym. None of the women LwD talked about visiting gyms. This may be because women LwD find walking itself to be sufficiently challenging due to their age and poor health:

*I can’t walk like I used to. I am also old, 50 [years of age]. How long can I live after that? Exercise is for good health like walking. If you keep sitting, you fall ill. Your body will suffer from a lot of ailments. That’s why I keep walking*. (14, woman LwD, age 50)

This woman made a conscious effort to go for a walk and, given her health and age, was the only form of PA in which she could regularly engage. Walking was her PA of choice: *‘There are many other exercises, but I don’t do them*’.

The ability to do vigorous exercise was associated with cultural beliefs regarding age. Age was perceived by participants to be associated with strength, which decreases with age, and so forms of PA that were felt to require substantial strength were deemed to be inappropriate. Walking, by contrast, was deemed acceptable because it was perceived to not involve much strength and required less energy.

#### Subtheme 2: weather/environment

The recommendation to walk outdoors regularly is not easy in places that have extreme weather, a polluted environment, or limited facilities (e.g. community parks). Some PLwD and their family framed their experience with engaging with PA in terms of unfavourable environmental aspects.

*In winter, it is difficult to walk in the morning as the wind is blowing and there might be more chance of getting sick with a cold. So, I [prefer to] walk in the evening. If I am not able to walk, then I rest for months until the winter passes.* (19, woman LwD, age 60)

Similarly, a family member of another woman LwD described this situation as follows:

*She will start to go for a walk when the weather changes [to more pleasant weather].* (13, husband of woman LwD).

Family members considered the weather itself to adversely affect health, so they did not encourage PLwD to walk in bad weather. People with access to outdoor spaces for PA tried to make modifications to sustain their PA. A woman LwD described how peer support allowed her to go for a walk before sunrise to avoid hot weather and how walking in a group created a safer environment in the dark. Yet, despite the weather proving to be a barrier for some PLwD, others were able to walk even in these conditions. This suggests that PLwD may engage in walking if it is part of their routine, irrespective of the weather.

Some people do not engage in outdoor activity due to a lack of suitable facilities.

*I walk along the road [pavement]. I do not have a garden, and there is not a park nearby. So, I can only walk on the road in the streets. If there is a park, then the person can even walk barefoot. In our area, there is no facility of this sort.* (23, man LwD, age 56)

Walking, although the preferred mode of PA for both genders, has its constraints. Even this simplest of activities can be hindered by environmental barriers.

### Theme 2: the role of family members in influencing PA

Not everyone in our sample received help from family members with their PA because expectations and the type of help needed varied with family roles, gender, age, and position. These cultural factors influenced both a person’s ability to engage in PA and whether help from a family member would be offered. Thus, an individual’s position within the family was an enabler for some people LwD to engage in PA and a barrier for others.

#### Subtheme 1: family roles and help offered to women

A cultural expectation of helping elders in the family was found to be an enabler for engaging in PA for these individuals. However, among women, such help was offered to those who were older, with no such help offered to younger women. Older women LwD accepted the help offered by family members and welcomed the idea of a family member accompanying them for a walk. Children, irrespective of their gender, accompanied older women LwD on their walk.

*For the past few days, my son has accompanied me for a walk. He offers to go for a walk, and we walk for about 30 minutes. Usually, he is busy at night, so we can’t go for a walk then. However, when he has time, he asks me to go for a walk with him, which I do.* (28, woman LwD, age 52)

Women seemed to prefer to rely on others (e.g. children) to encourage them to engage in PA. However, this sort of reliance could become a barrier if family members were unable to provide help consistently.

*My father’s health was not good, and she [her mother] wanted both of us to go for a walk with her. My brother would accompany her for a walk. My brother and I had a busy schedule because of which we could not accompany her at times.* (9, daughter of woman LwD)

As stated, women LwD were found to prefer to be reliant on others, suggesting that older women LwD were less likely to make an effort to engage in PA if internal sources within the family structure were not motivating them. However, help offered to older women by family members (e.g. facilitating peer meetings for older women) was found to be an enabler for these women to engage in PA.

The responsibilities associated with the role of older women LwD in the family were not found to be a barrier with PA engagement, but this was not the case for younger women LwD.

*There should be something [exercise] that a person can do at home. It is difficult to go for a walk on days when a person washes clothes or has a guest. A person gets tired.* (3, woman LwD, age 31)

Younger women LwD were found to lack the internal sources of support within the family structure and were culturally expected to have more responsibilities in terms of running the household and offering help to others in the family. Additionally, women LwD were limited by their husbands’ restrictions on outdoor activities, though these changed as they grew older.

*It is my opinion that I can do anything I like. I can easily go anywhere if I wish. Previously, I had to ask my husband before going outside the home, but now there is nothing like that at all. Either this is due to my age, that I am permitted, or it is known that I have to complete the task on my own. Earlier, my husband would take me along and bring me back himself, but now I can go anywhere. I have to inform him of my whereabouts though.* (24, woman LwD, age 45)

Overall, a woman’s age and position in the family could be a barrier to engaging in PA as these activities were dependent on the help offered and the permission of their husband, as head of the family, which reflects the cultural expectation of family hierarchy.

#### Subtheme 2: Family roles and help offered to men

Many men LwD stated that involving family members was unnecessary, implying that they considered it intrusive. If family members did not understand the importance of this cultural perception of men’s role and their ability to make their own decisions, it sometimes created problems in family interactions. Regarding the involvement of family members, a man LwD and his wife responded as follows:

*I do not need help from my family for exercise. I know what to do. It depends on the person if they want to start doing exercise.* (17, man LwD, age 55)

*He [her husband] will not go for a walk with me since I am a wife and not a friend. He likes doing these kinds of activities [walking] with his friends. Even if I ask him to come for a walk, he will not enjoy it. Going for a walk with me will be a duty for him as a husband.* (10, wife of man LwD)

She further added:

*We have not changed anything in the household to enable physical activity.* (10, wife of Man LwD)

Men preferred to be independent in making decisions about their tasks. This was elaborated upon by a family member (1, wife of man LwD) who described the relationship issues she encountered when telling her husband to go for a walk:

*I have been asking him to go with me for a walk after the [morning] prayers. We can walk for an hour or half an hour. He says, ‘I do not want to go for a walk’. My younger son insisted that his father goes for a walk with me. However, he said no. If we emphasised it, he would have become angry. He would verbally abuse [me] and start shouting.* (1, wife of man LwD)

Men LwD may not be willing to accept help as it may imply that they are not in control of the situation. The wife in this response attempted to influence her husband’s decision by making the decision herself to go for a walk, which disturbed the hierarchical interaction between husband and wife. However, women who were able to recognise this aspect of the culture used indirect strategies to influence their husbands. For example:

*I do encourage him by commenting on his looks. I think it helps if someone asks him what he has been doing to maintain his physical appearance. He feels happy when people notice that he has made an effort, and this encourages him more.* (10, wife of man LwD)

Similarly, another family member of a man LwD provides further information regarding family involvement:

*We do not plan anything else for the time slot that he [her father] allocates for walking.* (8, daughter of man LwD)

The comments above show that the specific nature of the help needed differs between genders. Family members wanting to offer help used methods that were more culturally acceptable to men LwD. Such methods included less intrusive involvement from family members and avoiding the use of direct suggestions by their wives.

### Theme 3: gender differences and cultural acceptability of an activity

Cultural acceptability of a behaviour is an essential enabler for engaging in PA.

#### Subtheme 1: cultural expectations regarding gender-specific activities

None of the women LwD or their family members in this study described an activity other than walking, whereas some men LwD mentioned running, playing cricket, and cycling. Some middle-aged men described taking part in more vigorous activities, which may be due to their health and ability to engage in such activities.

*I play cricket on the weekends. I play cricket in the morning before having my breakfast. I am a bowler.* (2, man LwD, age 37)

Cultural acceptance of behaviour was found to play a dominant role in men’s behaviour in this context. The efforts of men LwD to engage in vigorous activity (e.g. running on the road) were less acceptable in certain social settings because it was considered something unusual in that context.

*I do not run at a fast pace on the road. I run slowly. People will say look at me and make fun by asking, ‘Why is he running?’ Moreover, there is no park, no space; then I have to ride the bicycle at a fast pace to work.* (26, man LwD, age 57)

It may be unusual to see someone running on the road. However, riding a bicycle was common among working-class men. Performing vigorous activities in an unusual setting may give rise to unwanted attention.

#### Subtheme 2: gender-based activity

Some PLwD and their family members described engaging in same-gender peer support groups that enabled their PA. Not everyone had the option of outdoor walking. If they did, they were supported by same-gender peer-support walking groups. A woman LwD and her daughter-in-law describe their experiences below:

*I never miss a walk. I go for a walk with everybody. When we walk together [with peers], we don’t feel any difficulty, even if I am not well. There are three to four women, so we keep chatting while walking. There is this park nearby; we make two or three rounds of it. I started with one round, and now we do three rounds. We walk at a slower pace. We can’t walk fast.* (14, woman LwD, age 50)

She continued:

*Even if my family members are asleep, I go for a walk without them. My neighbours and I go for a walk after offering morning prayers. We gather one another and go together as a group. All of us have diabetes.* (14, woman LwD, age 50)

*Now she [mother-in-law] also encourages other women living in the neighbourhood to go for a walk.* (4, daughter-in-law of woman LwD)

Same-gender peer support was found to be culturally acceptable in an Islamic country. Women and the elderly reported the opportunity to gather in same-gendered groups in a garden or in their neighbourhood to go for a walk. These groups sustained walking because they were both only women’s groups and also a gathering of women who shared similar interests. Walking therefore became enjoyable because it involved having a group of friends of the same gender and served as a form of socialisation. However, this peer support was not available to everyone, particularly younger women LwD, some of whom expressed a desire to have someone to support their PA.

*If I have to go to the park, I will need to cross roads, which I am scared of after having ‘sugar’. My legs tremble, and I feel that my health is not the same as before. That is the reason I need someone to accompany me.* (3, woman LwD, age 31)

Most men LwD reported going for a walk alone, though some went with their peers.

*I try to have someone [a friend] accompany me when I go for a walk. In case I faint, I have someone to help in that situation. People go with me to offer morning prayers [in a mosque]. I feel the need to have someone accompany me when I go for a walk, which can also benefit them.* (16, man LwD, age 46)

Peer support for men LwD could be effective as it provided not only motivation but also a peer environment in which they could comfortably ask for help.

Similarly, a family member reported that men LwD were more motivated to walk with their peers.

*In the beginning, we had to force our dad to go for a walk. Now he has a walking buddy who lives nearby. So, he feels motivated to go for a walk. Either he calls him, or his friend calls him to ask if he wants to go for a walk. They go for a walk together.* (8, daughter of man LwD)

It appears that going for a walk becomes more sustainable as an enabler for men LwD if help comes in the form of companionship. This companionship can be considered a form of social support.

## Discussion

This study adds to the understanding of culture as an important factor in helping PLwD incorporate purposive, deliberate PA into their daily lives. Culture, based on the findings here, can be conceived as the important role played by gender, age, and place in the family hierarchy which underpins many of the findings. In addition, the physical and socio-economic context are other important elements in shaping what PA is possible for a person LwD and how a household member may help or hinder their engagement in PA.

The guidelines for PA in Pakistan are similar to Western guidelines, which are more suited to individualistic cultures than collectivistic cultures. Our findings suggest that these guidelines may not be as effective in leading to behaviour change in a society in which family and cultural roles are central. Moreover, even in the same culture, one size does not fit all. Our findings suggest that people of different genders and ages are subject to different limitations, which provides some with more opportunities than others to engage in PA. Furthermore, our findings suggest that a family member can only support someone LwD with their PA if their help does not conflict with conventional norms of the hierarchical family structures embedded within Pakistani culture.

Walking is preferred by people from SA cultural backgrounds, whether residing in their native countries such as Pakistan [7, 42] or in the West [18, 28]; this is supported by the current study. Previous studies have reported reasons for not engaging in PA similar to some of the reasons noted here, including household routines; difficulty finding the time [19, 27, 43]; cultural limitations such as requirements for a same-gender environment, which is unavailable in gyms [28]; a lack of places to go for a walk [27, 44]; and the absence of a family member to walk with [42]. However, previous studies [45, 46] have not acknowledged the importance of the familial context in which people live, resulting in gaps in knowledge that may account for differences among study findings in SA communities versus Western communities. This study showed that opportunities for PA vary considerably across gender, age, and family role. PA options are guided by the cultural rules governing expected and appropriate behaviours. These behaviours are guided by hierarchical structures that inform the power dynamics among family members, influencing decisions about whether or not to engage in PA.

The cultural expectation that support will be offered to the elderly, for example, acts as enabler for PA such as walking. It is a sign of respect to make sure that they, especially women, have their needs met, which is associated with the description of a good son or daughter in this culture [23, 26, 34]. Additionally, in Pakistan, like other Muslim countries, adult children have a religious obligation to show respect by expressing kindness towards and obeying their parents [47].

Similar to the previous literature, this study suggests that although family involvement can be helpful for encouraging PA in PLwD [45], it can also be a barrier [46]. Women can become more dependent on others for being physically active. It can hinder older women LwD regarding their ability to engage independently in PA. Nevertheless, it is important to understand that adult children cannot directly stop offering support, as older adults may see this as a sign of disrespect and a lack of care [23, 47]. Family involvement could be used in the initial phases and later shifted to peer support, where women help women LwD to be more independent in regard to their lifestyle decisions.

These cultural beliefs and religious obligations may support older women, but they may act as a barrier for younger women who are culturally expected to offer support to other vulnerable family members. Their agency is influenced by the cultural interpretation of roles and responsibilities within the family. There appears to be a strong preference in SA communities to fulfil the expectations associated with these roles, as supported by Majeed-Ariss et al.’s [48] study of first- and second-generation Pakistani women LwD in the United Kingdom. They found that British Pakistani women LwD wanted to maintain their identity in their families. First-generation Pakistani women who were homemakers wanted to fulfil their role of looking after others rather than putting themselves first. Identity within the family is strongly supported by PLwD in SA culture. Therefore, medical recommendations must work with cultural expectations regarding societal and familial roles to avoid conflict with everyday behaviour.

Men benefit more from engaging in PA, either with their peers or alone. The cultural role of men is to be independent; they must be in control and able to make their own decisions [23, 26, 34]. Medical recommendations around family members’ involvement must encourage an approach that does not conflict with these traditional roles.

### Implications

Lifestyle adjustment is not about addressing just one aspect of the cultural environment (e.g. health beliefs); rather, a large number of factors are at play: family role, gender, and age are three crucial factors shaping SA communities. These factors inform the link between agency and control among family members and influence lifestyle change.

Medical recommendations often involve activities that do not fit with women’s schedules or cultural expectations. One way to offer support for self-care behaviour to younger women LwD could be by, in future studies, recording their steps through wearable technology or mobile applications to obtain information on the amount of activity performed over the course of a day [49]. Information on the amount of energy expended during household chores could then be used to make recommendations regarding the duration or intensity of these activities to attempt to match the current activity-related recommendations for PLwD.

Campaigns should be developed based on an understanding of these home-related activities, and younger women should be encouraged to engage in these. Younger women should be encouraged to see these activities as forms of exercise. It is important that doctors/physicians recognise them as valid forms of PA; otherwise, encouraging women to recognise them as such will fail**.** These approaches would not only provide options for PA but would also give the women agency to make decisions related to healthcare recommendations.

Men’s cultural roles are centred on being independent and strong, and they make many decisions within the family [24]. Control is less appreciated when it comes from people lower in the family hierarchy [24].

### Strengths and limitations of this study

Considering these findings, it is critical to be mindful of the limitations of the methods used. Semi-structured interviews were used to elicit the perspectives of participants. However, the details gathered through interviews and an exploration of participant behaviours on site led to the understanding that the choices around lifestyle changes observed in a natural setting could provide a more detailed account.

Another limitation of the study is that no clinical data or records were obtained. Above all, no data on levels of glycaemia were collected and future research should aim to combine qualitative interview data with biological markers of self-care and disease status.

The sample reflected people from lower- or middle-income socioeconomic backgrounds, limiting the findings to these demographics. Given the need to move away from a middle-class bias in research, and target what is sometimes referred to as underserved groups in society, this is a strong positive aspect.

## Conclusions

The guidelines for PA implemented for PLwD in Pakistan are the same as those published in Western high-income countries, which regard PLwD as responsible for their own health and as having options readily available to them. However, that is not the case for every member of SA families. Unlike in Western contexts, societal norms, cultural expectations, and health beliefs that inform the behaviour of PLwD in SA communities are given little consideration, possibly explaining the less successful health outcomes observed in SA communities. Doctors’ recommendations could be more family-centred focusing on the options available to specific families and family members, given their age, gender, and family role, to help them successfully engage in PA to manage their condition.

Medical teams and the Ministry of Health in Pakistan should acknowledge the cultural forces and constraints faced by PLwD. Furthermore, they should draw on the norms of Pakistani populations and groups to enable behaviour change within the family and social context. Health promoters and educators in government and clinical settings need to work with, rather than ignore, cultural values and norms regarding age, gender, and role in families, as well as individual health beliefs.

## Data Availability

Research data are not shared.

## Abbreviations

*LMIC*: Low- or middle-income countries
*LwD*: Living with diabetes
*NCD*: Non-communicable disease
*PA*: Physical activity
*PLwD*: People living with type 2 diabetes
*SA*: South Asian
*T2DM*: Type 2 diabetes mellitus
*WHO*: The World Health Organization
